# Mediterranean Diet Adherence and Its Relationship to Metabolic Markers and Body Composition in Portuguese University Students

**DOI:** 10.3390/nu15102330

**Published:** 2023-05-16

**Authors:** Sofia Lopes, Tatiana Fontes, Regina Menezes, Luís Monteiro Rodrigues, Cíntia Ferreira-Pêgo

**Affiliations:** CBIOS—Universidade Lusófona’s Research Center for Biosciences and Health Technologies, Av. Campo Grande 376, 1749-024 Lisbon, Portugal; f6775@ulusofona.pt (S.L.); f6776@ulusofona.pt (T.F.); regina.menezes@ulusofona.pt (R.M.); monteiro.rodrigues@ulusofona.pt (L.M.R.)

**Keywords:** Mediterranean diet, university students, body composition, adipose tissue, metabolic markers

## Abstract

Background: Transitioning to university involves several changes, which might affect dietary habits. The present study aimed to assess the potential relationships involving adherence to the MedDiet, body composition, and metabolic markers within a Portuguese university sample. Methods: A cross-sectional study involved 70 participants, 52 women, and 18 men (23.00 ± 7.00 years old and a BMI of 21.99 ± 2.79 kg/m^2^). The average MedDiet adherence of participants was 9.23 points, as evaluated by the 14 point validated questionnaire, with classifications of low and high (under or over 9 points, respectively). Body composition was assessed using X-ray dual densitometry (DXA), and metabolic markers were collected from capillary blood. Results: Statistically significant differences in HDL cholesterol and the total/HDL cholesterol ratio were found between groups. Lower levels (*p* < 0.05) of visceral (VAT) and subcutaneous adipose tissue (SAT), BMI, and waist circumference were found in the higher MedDiet adherence group. Those measures were negatively correlated (*p* < 0.05) with the adherence scores to the MedDiet. Conclusion: Higher adherence to MedDiet seemed to have a favorable and important impact on lipid profiles, primarily HDL-c. A positive relationship between MedDiet adherence and body composition distribution was also described, mostly due to the influence of higher adherence to MedDiet at lower levels of VAT and SAT in Portuguese university students.

## 1. Introduction

The Mediterranean diet (MedDiet), commonly associated with southern Europe and Mediterranean areas [1], is characterized by high consumption of fresh fruits, vegetables, extra-virgin olive oil, nuts, whole grains, and legumes, moderate consumption of fish and dairy products (mainly fermented), and low intake of red meat or processed foods [2,3,4]. These characteristics were recorded on the representative list of the Intangible Cultural Heritage of Humanity (UNESCO) in 2021 [5]. MedDiet has been referred to as effective in controlling obesity, a pandemic currently challenging health systems worldwide [3,4,6]. In this line, it has been associated with a risk reduction for multiple chronic diseases, including cardiovascular, metabolic, neurodegenerative, and some types of cancer, among others [2,6,7,8,9,10]. This might result from the high anti-inflammatory activity of MedDiet components related to the consumption of short-chain fatty acids, polyunsaturated fatty acids, (poly)phenols [11,12], and antioxidants [1,9]. In spite of several mechanisms that have been suggested to explain these impacts, many doubts still persist. Without doubt, MedDiet has been clearly linked to an improved quality of life [3], influencing both physical and mental well-being [3], and consequently decreasing morbidity and mortality of all causes [2,13]. These beneficial effects might also be related to and influenced by the positive relationship between increased MedDiet adherence and lower body composition, as higher adherence to MedDiet has been associated with a decrease in fat mass (FM) [10] and an increase in fat-free mass (FFM) [14]. Studies have shown a positive impact of the MedDiet on metabolic health, likely due to its high consumption of plant-based foods, which are high in antioxidants, fiber, and other nutrients [1,9]. The MedDiet is associated with lower inflammation [15], lower blood pressure, lower levels of total cholesterol and low-density lipoprotein (LDL) [16], and higher levels of high-density lipoprotein (HDL) [17]. Overall, the research data points to the possibility that following the MedDiet can improve metabolic indicators and be beneficial in the prevention and treatment of many chronic diseases [15,18].

The lifestyle shifts for young adults when entering university are known to determine multiple changes [2,10,13], including an increase in FM and Body Mass Index (BMI) [14,19]. Currently, the eating habits of college students resemble a Western eating pattern since there is a higher consumption of “fast”, processed, and industrialized food and products with high energy content [10,13], which might directly relate to mass gain and metabolic risk [2,13,19].

In this context, we decided to investigate a group of university students at a Portuguese university where the MedDiet is a reference to better understand potential relationships among MedDiet adherence, body composition, and relevant metabolic markers.

## 2. Materials and Methods

### 2.1. Study Design and Population

A cross-sectional observational study was designed. Recruitment took place on the University Campus of the Lisbon Metropolitan Area between January 2022 and February 2023, resulting in a final sample of 70 individuals of both sexes, aged between 18 and 39 (23.00 ± 7.00) years old, who were pursuing a bachelor’s, a master’s, or a doctoral degree. Non-inclusion criteria were being underage, regular consumption of any medicines, being (or suspecting to be) pregnant and/or breastfeeding, being/feeling sick, and not being a university student. All participants were Caucasian, and data were collected by trained nutritionists in a face-to-face interview. All participants provided signed, informed written consent. Procedures followed all principles of good clinical practice adopted for human research studies, as described in the Helsinki Declaration and its further amendments [20]. The study was previously approved by the Institutional Ethics Committee (CE.ECTS/P05-21).

### 2.2. Participants’ Characterization and Instruments

General characterization data, such as sex, age, area of residence (urban or rural), and place of residence according to the division of the Nomenclature of Territorial Units for Statistics (NUTS) II [21], were collected for all participants. Lifestyle data such as smoking or other habits, medication, supplementation, hours, and quality of sleep according to the Pittsburgh sleep quality index study [22], as well as other personal and familiar information of interest, were also registered. Physical activity was evaluated using the short version of the validated International Physical Activity Questionnaire (IPAQ) [23,24]. Mass was measured by an electronic scale [0.1 kg (0.1–200 kg) accuracy], wearing light clothing and no shoes. Height was a self-reported variable used to calculate Body Mass Index (BMI) according to Quetelet’s formula [body mass (kg)/height (m)^2^] [25]. The waist circumference was measured using a tape at the midpoint between the last rib and the iliac crest after full exhalation of air. Dual-energy X-ray absorptiometry (DXA) (Lunar Prodigy Advance–General Electric Healthcare^®^, Chicago, IL, USA) was used to measure bone mass, fat mass, lean mass, tissue mass, fat-free mass, total mass, and visceral and subcutaneous adipose tissue. Before each whole-body scan, the DXA was calibrated according to the manufacturer’s instructions using a standard calibration block. All measurements were collected under the same atmospheric conditions by the same researcher. To assess MedDiet adherence, participants were given a validated 14 item questionnaire [26,27]. This consisted of 12 questions on food consumption frequency and 2 questions on food intake habits considered characteristic of the Mediterranean diet. Each question was scored 0 (if the condition was not met) or 1 (if the condition was met). The final score ranged from 0 to 14 points. Using this score, MedDiet adherence was classified into two categories: low (<9 points) and high (≥9 points). A portable automatic testing device, the LINX DUO (Menarini Diagnostics^®^), was used to assess metabolic markers such as glycated hemoglobin (HbA1c), the lipid profile (triglycerides, total cholesterol, HDL), and blood glucose. Taking these variables into consideration, LDL cholesterol, very-low-density lipoprotein (VLDL), total cholesterol/HDL ratio, and non-HDL cholesterol were also quantified. For the blood extraction process, a puncture device (OneTouch Ultra Soft with Microlet 23 gauge lancet) was used. An automatic monitor (Tensoval–HARTMANN^®^) was used to measure the three components of blood pressure: systolic, diastolic, and heart rate. Before the measurement, the volunteer rested for 5 min and sat upright with the back supported, both feet flat on the floor, and the upper arm supported at heart level. All measurements were taken in the non-dominant arm, with the middle of the cuff positioned with its midsection over the brachial artery. Measurements were performed with at least 12 h of fasting (including no water, caffeine sources, diuretics, and/or alcoholic beverage consumption) and no exercise in the previous 24 h to ensure proper hydration conditions. All participants were also asked to empty their bladders prior to measurements.

### 2.3. Statistical Analysis

Statistical analysis was performed using IBM SPSS Statistics version 22 (SPSS Inc., Chicago, IL, USA). Parametric tests were used when the sample presented a normal distribution, and non-parametric tests when the sample presented a non-normal distribution. The Kolmogorov–Smirnov normality test was used when *n* > 50 and the Shapiro–Wilk normality test when *n* ≤ 50. Nominal variables were shown as percentages (frequencies) and continuous variables as mean (standard deviation, SD) or median (interquartile range, IQR), depending on the type of variable. For the comparison between two categorical variables, the chi-squared, Fisher’s exact, and Monte Carlo tests were used as appropriate. The Student’s *t*-test and Mann–Whitney U test were used between categorical and scale variables. The Pearson or Spearman’s rank correlation coefficients were used to examine the relationship between the MedDiet score, metabolic indicators, and body composition values. All statistical tests were two-tailed, and the significance level was set at *p* < 0.05.

## 3. Results

The participants in our study had MedDiet adherence scores ranging from 5 to 13, with a mean score of 9.23. Twenty-three participants out of seventy (32.86%) presented low adherence, and forty-seven (67.14%) had high adherence. The general characteristics of the study population are shown in Table 1. The mean age was 23.00 ± 7.00 years old, and the average BMI was 21.99 ± 2.79 kg/m^2^. The mean height was 1.67 ± 0.08 m, and the median body mass was 59.40 ± 14.00 kg. Participants with low adherence showed significantly higher values of BMI and waist circumference.

Metabolic markers for each MedDiet adherence category are presented in Table 2. Low-adherence individuals had significantly lower values of HDL cholesterol and higher values of the total cholesterol/HDL ratio compared to participants in the high-adherence categories. Noteworthy, high-adherence individuals presented lower glycemia values compared to the other group; however, these results were not statistically significant (*p* = 0.057).

Table 3 shows body composition distribution according to MedDiet adherence categories. Participants with lower adherence had significantly higher Subcutaneous Adipose Tissue (SAT) content (1076.61 ± 603.05 cm^3^ vs. 751.58 ± 379.03 cm^3^) and Visceral Adipose Tissue (VAT) content (267.00 ± 334.00 cm^3^ vs. 106.00 ± 155.00 cm^3^), compared to individuals with higher adherence to the MedDiet.

Average MedDiet adherence scores according to demographic characteristics can be observed in Table 4. Out of a range of 0–14 points, the mean adherence scores for men and women were 8.78 and 9.38, respectively. There were no significant differences, but participants in the 2nd year of their bachelor’s course presented a mean of 10.20 points, higher than the rest of the study years (*p* = 0.051).

**Figure 1:**
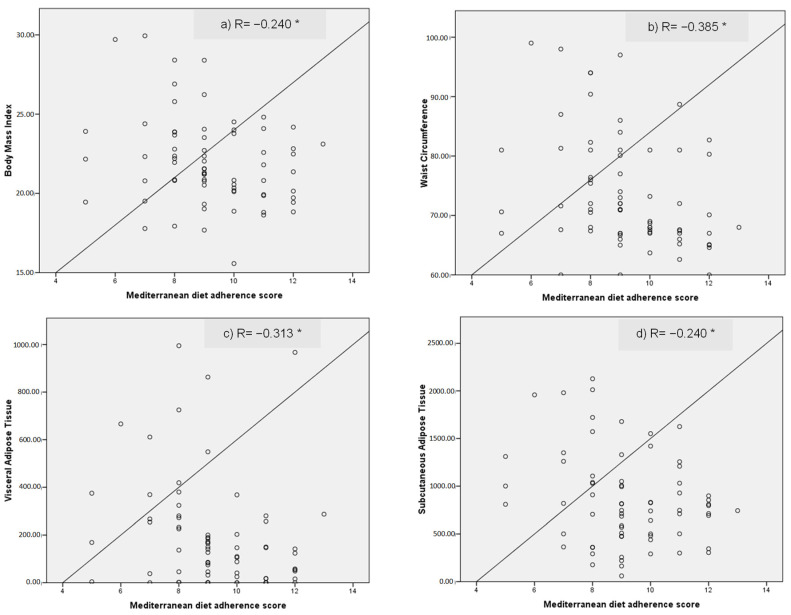
Scatter charts evaluated the correlation (R) between Mediterranean Adherence Score and (**a**) Body Mass Index; (**b**) Waist Circumference; (**c**) Visceral Fat Tissue and (**d**) Subcutaneous Fat Tissue. * Statistical significance *p* ≤ 0.05.

Mediterranean diet adherence scores, BMI, anthropometric measurements, and fat tissue correlations are shown in Figure 1 as scatter plots. Higher adherence scores significantly correlate with lower values of BMI, waist circumference, VAT, and SAT.

Table 5 shows the agreement with the dietary recommendations based on the 14 item MedDiet adherence questionnaire according to sex, course, house living, and income. Low adherence was observed for each of the items. Additionally, volunteers enrolled in a course not related to health sciences presented a higher percentage of agreement with a portion of legumes than those studying a health sciences-related course (*p* = 0.002). Participants living with parents had higher adherence scores than those living with friends. Individuals with monthly incomes between EUR 1000 and 3000 tended to comply more with the MedDiet adherence criteria than other income levels.

## 4. Discussion

The present study was designed to provide a more thorough assessment of the relationship between adherence to MedDiet, body composition, and metabolic markers in a cohort of healthy Portuguese university students. Recent surveys have produced slightly different results regarding the prevalence of MedDiet adherence [28]. It appears that adherence is largely influenced by cultural heritage, but it is unclear why adherence is lost over time [28]. The use of different questionnaires and cut-off points to measure adherence might contribute to this result’s variability [3]. The globalization of markets has led to a “Westernization” of the overall European diet, with younger generations leaving traditional dietary patterns [28] as they have more access to non-Mediterranean food groups such as animal fats, vegetable oils (other than olive oil), sugar, and red meat [3,28]. Overall, studies [3,29] show low rates of adherence to the MedDiet, particularly among young people who tend to move away from healthy lifestyle habits. This seems to be influenced by emotional, physiological, and environmental changes [30] experienced during this life period, leading to changes in their dietary patterns [31], including an increase in fast food consumption [29].

Regarding metabolic markers, the HDL cholesterol and total cholesterol/HDL ratio were statistically more favorable in those students with higher MedDiet adherence. Low HDL and high LDL cholesterol levels are known to be the main determinants of cardiovascular risk [32,33]. The MedDiet has been suggested to improve cardiovascular health due to the high omega-3 and omega-6 content of extra-virgin olive oil and nuts [17,32]. Healthier levels of other lipid profile variables, such as triglycerides, LDL, and non-HDL cholesterol, were observed in the higher adherence category, although differences were not statistically significant. These differences have been previously observed and may be explained by the higher consumption of water-soluble fiber associated with this dietary pattern [14,32].

Concerning body composition distribution, our study has shown that higher MedDiet compliance led to significantly lower levels of VAT and SAT. Similar results have been published [14,34], but the evidence regarding the relationship between MedDiet and VAT is still not clear [3]. Although both VAT and SAT contribute to overall body fat mass, they have distinct characteristics [35,36]. VAT surrounds the abdominal organs, including the liver, pancreas, and gut, and is more metabolically active than SAT, rather than being associated with insulin resistance, inflammation, and other metabolic disorders [37]. On the other hand, SAT is located directly beneath the skin and serves as a primary energy reserve for the body [36]. Unlike VAT, however, SAT does not seem to be associated with metabolic dysfunction and disorders [37]. Participants who are living with their parents seem to have higher adherence to the MedDiet. This may be due to the fact that older people generally demonstrate greater adherence levels as compared to younger individuals [28]. Given that family and environment are crucial in shaping dietary habits and food preferences [38], parents, grandparents, and older siblings have a significant impact on establishing “healthy” eating habits in their children [39]. In the case of the MedDiet, family-based food traditions and practices could be a vital element in encouraging adherence to this dietary pattern [40]. Participants with low socioeconomic levels seem to follow fewer items related to healthier MedDiet adherence than those with a higher socioeconomic level. It may be that individuals with lower socioeconomic status tend to have less accessibility to fresh fruits and vegetables, which are usually more expensive than processed foods [31]. This can make it difficult to follow a diet rich in these foods, such as MedDiet. The results of our study indicate that the consumption of olive oil was statistically higher among participants with low incomes, and similar results were found for all the groups analyzed (EUR < 1000, EUR 1000–3000, and EUR > 3000). The countries situated in the Mediterranean region, including Portugal, provide olive oil at comparatively lower prices, which may further explain its increased usage among these participants [3]. Our correlation analysis seems to be in line with recent observations suggesting that higher adherence to the MedDiet was statistically related to lower values of VAT and SAT [14,32,41], and with lower BMI and waist circumference [42,43], as seen before. These observations may be explained by the composition of the typical Mediterranean diet, which includes many foods, such as fruits and vegetables, with low energy densities and high water and fiber contents [42]. These foods increase the feeling of satiety, as they require more chewing and take longer to digest than foods with high energy density, which can lead to reduced energy intake, resulting in weight loss [43]. The adoption of an unhealthy lifestyle, similar to a more Western dietary pattern, is common during this time of transition, as previously mentioned [2]. A Western-style diet has been linked to the development of several non-communicable chronic diseases [2,44], such as obesity, cardiovascular disorders, Type II diabetes mellitus, and even mental health, as a result of fatigue and a lack of energy caused by a lack of essential nutrients [45]. Even though the young participants analyzed in our study appeared to be in “good health”, our results showed that those in the low MedDiet adherence group had statistically unhealthier values for metabolic markers associated with cardiovascular health and for body composition associated with metabolic dysfunction and disorders. Although young people (generally) appear to have strong health and resistance to disease conditions, the effects of poor adherence to this dietary pattern can be cumulative [45], resulting in poor long-term quality of life and the possible development of the aforementioned health problems [45]. Thus, it is important to promote healthy eating habits in younger adult populations, including increased adherence to MedDiet. This can be achieved through nutrition education, beginning by promoting healthy eating environments within colleges and universities themselves [28].

To the best of our knowledge, this is the first study developed in a university student population demonstrating that higher adherence to the MedDiet was related to more favorable body composition distribution and metabolic marker levels. Our conclusions are well supported by the assessment technologies used. DXA is one of the most specific methods of body composition assessment [46]. Metabolic markers were assessed using a minimally invasive method.

Some limitations should be assumed in the present study. The reduced sample size and the cross-sectional design do not allow the establishment of a causal effect. Self-reported height may be a limitation, as it affects BMI calculations. In our study, the values were confirmed by comparison with the values registered on the participants citizen card. Several studies have already demonstrated the reliability and accuracy of using the values from a citizen’s card to obtain some anthropometric measurements [45]. Another limitation can be attributed to the fact that the validated questionnaire on MedDiet adherence is not adapted to the gastronomic differences of each country [3]. As an example, high consumption of vegetable soup has been identified in Portugal; however, this is not specifically evaluated in the questionnaire. Further, the question regarding wine consumption (seven glasses/week) may be inappropriate for the age group investigated since young adults usually do not drink wine to the same extent as older adults. Additionally, the binary scoring system for different food items does not allow for the assessment of the degree of proximity to the optimal consumption of a particular item [47].

## 5. Conclusions

The present study demonstrated for the first time that higher MedDiet adherence seems to lead to favorable metabolic marker levels in young Portuguese university students, showing healthier values of HDL-c and the TC/HDL-c ratio and also being related to healthier body composition due to the reduced levels of SAT and VAT. Poor adherence to MedDiet may have future negative impacts on health and quality of life and may affect physical and mental well-being. Given that this study presented a cross-sectional design, the implementation of programs that encourage adherence to MedDiet in young adults is important to maybe help prevent some non-communicable chronic diseases and improve quality of life in the long term.

## Figures and Tables

**Table 1:** Sociodemographic characteristics of Portuguese university students according to categories of Mediterranean diet adherence.

	Total Population (*n* = 70)	Low Adherence (*n* = 23)	High Adherence (*n* = 47)	*p*-Value
**Sex, % (*n*)**				
Male	25.70 (18)	39.10 (9)	19.10 (9)	0.072 ^c^
Female	74.30 (52)	60.90 (14)	80.9 (38)	
**Age, years**	23.00 (7.00)	24.00 (7.00)	23.00 (7.00)	0.598 ^b^
**Height, m**	1.67 (0.08)	1.68 (0.72)	1.66 (0.85)	0.500 ^a^
**Mass, kg**	59.40 (14.00)	63.80 (20.80)	57.10 (13.60)	0.138 ^b^
**BMI, kg/m^2^**	21.99 (2.79)	23.10 (3.34)	21.44 (2.32)	0.018 ^a^
**Waist** **Circumference, cm**	70.95 (14.00)	76.00 (16.50)	68.00 (7.30)	0.004 ^b^
**Liquid** **Ingestion, L/day**	2.00 (1.00)	2.00 (1.00)	2.00 (1.00)	0.749 ^b^
**Monthly Family Income, % (*n*)**				
Under EUR 1000	8.60 (6)	8.70 (2)	8.50 (4)	1.000 ^d^
EUR 1000–3000	65.70 (46)	65.20 (15)	66.00 (31)	
EUR > 3000	25.70 (18)	26.10 (6)	25.50 (12)	
**Smoking Status, % (*n*)**				
Smoker	11.40 (8)	17.40 (4)	8.50 (4)	0.564 ^d^
Ex-Smoker	11.40 (8)	13.00 (3)	10.60 (5)	
Non-Smoker	77.10 (54)	69.60 (16)	80.90 (38)	
**Academic course, % (*n*)**				
Health	62.90 (44)	60.90 (14)	63.80 (30)	0.810 ^c^
Other	37.1 (26)	39.10 (9)	36.20 (17)	
**Home Living, % (*n*)**				
Parents’ home	48.60 (34)	56.50 (13)	44.70 (21)	0.590 ^c^
With other students	17.10 (12)	13.00 (3)	19.10 (9)	
Partner	18.60 (13)	21.70 (5)	17.00 (8)	
Alone	15.70 (11)	8.70 (2)	19.10 (9)	

Data are expressed as a percentage (*n*), mean (SD), or median (IQR) for categorical and continuous variables, as appropriate. *p*-values for group comparisons were tested by ^a^ Student’s *t*-test/ ^b^ Mann–Whitney U test or ^c^ chi-squared/ ^d^ Monte Carlo test, as appropriate. Abbreviations: BMI, Body Mass Index.

**Table 2:** Metabolic markers according to categories of Mediterranean diet adherence.

	Total Population (*n* = 70)	Low Adherence (*n* = 23)	High Adherence (*n* = 47)	*p*-Value
**Hemoglobin A1c, %**	5.23 (0.40)	5.19 (0.39)	5.26 (0.40)	0.530 ^a^
**Triglycerides, mg/dL**	93.50 (68.00)	89.00 (73.00)	98.00 (70.00)	0.822 ^b^
**Total** **Cholesterol, mg/dL**	169.24 (32.09)	167.30 (36.57)	170.19 (30.04)	0.726 ^a^
**HDL, mg/dL**	60.77 (15.61)	53.87 (9.90)	64.15 (22.00)	0.009 ^a^
**Glucose, mg/dL**	89.50 (15.00)	94.00 (14.00)	88.00 (12.00)	0.057 ^b^
**LDL, mg/dL**	82.00 (27.45)	90.50 (40.00)	81.00 (28.00)	0.210 ^a^
**VLDL, mg/dL**	19.00 (13.00)	17.50 (14.00)	20.00 (14.00)	0.523 ^a^
**TC/HDL ratio, mg/dL**	2.75 (0.84)	3.06 (1.07)	2.65 (0.74)	0.017 ^a^
**Non-HDL, mg/dL**	105.00 (30.00)	111.50 (44.00)	105.00 (26.00)	0.460 ^a^
**Systolic Blood Pressure, mm Hg**	110.00 (12.06)	113.13 (10.57)	108.47 (12.55)	0.130 ^a^
**Diastolic Blood Pressure, mm Hg**	70.50 (14.00)	70.00 (13.00)	71.00 (15.00)	0.131 ^b^
**Heart Rate, bpm**	68.00 (14.00)	66.00 (13.00)	69.00 (17.00)	0.381 ^b^

Data are expressed as a mean (SD) or median (IQR). *p*-values for group comparisons were tested by ^a^ Student’s *t*-test or ^b^ Mann–Whitney U test, as appropriate; Abbreviations: HDL, High-Density Lipoprotein; LDL, Low-Density Lipoprotein; VLDL, Very-Low-Density Lipoprotein; TC/HDL, Total Cholesterol/High-Density Lipoprotein; NHDL, Non-High-Density Lipoprotein; bpm, beats per minute.

**Table 3:** Body composition distribution according to categories of Mediterranean diet adherence.

	Total Population (*n* = 70)	Low Adherence (*n* = 23)	High Adherence (*n* = 47)	*p*-Value
**Total BMD, %**	1.94 (0.24)	1.86 (0.23)	1.98 (0.24)	0.060 ^a^
**Total BMC, %**	3.88 (0.33)	3.81 (0.29)	3.91 (0.35)	0.229 ^a^
**Fat Mass, %**	27.88 (6.90)	28.76 (7.88)	27.45 (6.41)	0.460 ^a^
**Lean Mass, %**	68.23 (6.73)	67.41 (7.72)	68.63 (6.24)	0.481 ^a^
**Fat-Free Mass, %**	72.11 (6.89)	71.22 (7.88)	72.54 (6.39)	0.455 ^a^
**VAT, cm^3^**	141.50 (238.00)	267.00 (334.00)	106.00 (155.00)	0.005 ^a^
**SAT, cm^3^**	858.37 (485.16)	1076.61 (603.05)	751.58 (379.03)	0.008 ^b^

Data are expressed as a mean (SD) or median (IQR). *p*-values for group comparisons were tested by ^a^ Student’s *t*-test or ^b^ Mann–Whitney U test, as appropriate. Abbreviations: BMD, Bone Mineral Density; BMC, Bone Mineral Content; VAT, Visceral Adipose Tissue; SAT, Subcutaneous Adipose Tissue.

**Table 4:** Mediterranean diet adherence scores by demographic characteristics.

	Mean (SD)	*p*-Value ^a^
**Gender**		
Male	8.78 (1.63)	0.221
Female	9.38 (1.85)	
**Academic course**		
Health	9.25 (1.87)	0.898
Other	9.19 (1.72)	
**Student Worker**		
Yes	9.21 (1.98)	0.904
No	9.26 (1.59)	
**Grade Level**		
Bachelor’s	9.33 (1.86)	0.543
Master’s	8.82 (1.43)	
PhD	9.57 (2.30)	
**Year Coursing—Bachelor’s**		
1st	8.33 (1.75)	0.051
2nd	10.20 (2.17)	
3rd	9.83 (1.63)	
4th	8.36 (1.86)	
**Type of housing**		
Family	9.12 (1.84)	0.620
Friends/Partner/Alone	9.33 (1.79)	
**Residence**		
Urban	9.07 (1.71)	0.104
Rural	10.00 (2.13)	
**Monthly Family Income**		
Up to EUR 1000	8.67 (1.51)	0.697
EUR 1000–3000	9.33 (1.71)	
EUR > 3000	9.17 (2.15)	
**Smoking Status. %** (***n***)		
Smoker	8.25 (1.74)	0.224
Ex-Smoker	9.00 (2.00)	
Non-Smoker	9.41 (1.74)	
**PAL**		
Low	9.18 (2.27)	0.413
Medium	9.47 (1.78)	
High	8.76 (1.25)	

Data are expressed as mean (SD); ^a^ statistical significance *p* ≤ 0.05. Abbreviations: PAL, Physical Activity Level.

**Table 5:** Agreement with the recommendations based on each item of MEDAS-14 by category.

	Recommendation	Compliance by Sex			Compliance by Course			Compliance by Living Arrangement			Compliance by Income Level			
		Men	Women	*p*-Value ^a^	Health	Other	*p*-Value ^a^	Friends	Family	*p*-Value ^a^	EUR < 1000	EUR 1000–3000	EUR > 3000	*p*-Value ^a^
1. Do you use olive oil as a main culinary fat?	Yes	94.40	94.20	0.973	95.50	92.30	0.584	100.00	88.90	0.045	100.00	93.50	94.40	0.811
2. How much olive oil do you consume in a given day (including the oil used for frying. salads. out-of-house meals. etc.)?	≥4 tbsp	22.20	34.60	0.329	36.40	23.10	0.247	38.20	25.00	0.233	83.30	28.30	22.20	0.015
3. How many vegetable servings do you consume per day? (1 serving: 200 g consider side dishes as half a serving))	≥2 (≥1 portion raw or as a salad)	72.20	73.10	0.944	72.70	73.10	0.975	67.60	77.80	0.341	100.00	76.10	55.60	0.074
4. How many fruit units (including natural fruit juices) do you consume per day?	≥3	61.10	48.10	0.340	50.00	53.80	0.756	55.90	47.20	0.469	16.70	58.70	44.40	0.121
5. How many servings of red meat. hamburger. or meat products (ham. sausage. etc.) do you consume per day? (1 serving: 100–150 g)	<1	66.70	86.50	0.062	81.80	80.80	0.913	76.50	86.10	0.300	100.00	80.40	77.80	0.459
6. How many servings of butter. margarine. or cream you consume per day? (1 serving: 12 g)	<1	72.20	80.80	0.446	77.30	80.80	0.730	73.50	83.30	0.318	66.70	71.70	100.00	0.035
7. How many sweet or carbonated beverages do you drink per day?	<1	88.90	92.30	0.655	90.90	92.30	0.840	88.20	94.40	0.354	66.70	93.50	94.40	0.076
8. How much wine do you drink per week?	≥7 glasses	5.60	7.70	0.762	6.80	7.70	0.891	8.80	5.60	0.596	0.00	4.30	16.70	0.177
9. How many servings of legumes do you consume per week? (1 serving: 150 g)	≥3	66.70	59.60	0.596	47.70	84.60	0.002	44.10	77.80	0.004	66.70	58.70	66.70	0.809
10. How many servings of fish or shellfish do you consume per week? (1 serving of 100–150 g of fish or 4–5 units or 200 g of shellfish)	≥3	27.80	73.10	0.001	72.70	42.30	0.012	70.60	52.80	0.126	33.30	67.40	55.60	0.229
11. How many times per week do you consume commercial sweets or pastries (not homemade). such as cakes. cookies. biscuits. or custard?	<3	88.90	67.30	0.076	72.70	73.10	0.975	61.80	83.30	0.043	33.30	78.30	72.20	0.066
12. How many servings of nuts (including peanuts) do you consume per week? (1 serving 30 g)	≥3	55.60	55.80	0.987	56.80	53.80	0.809	47.10	63.90	0.157	33.30	63.00	44.40	0.207
13. Do you preferentially consume chicken. turkey. or rabbit meat instead of veal. pork. hamburger. or sausage?	Yes	61.10	73.10	0.340	77.30	57.70	0.084	79.40	61.10	0.095	50.00	71.70	72.20	0.535
14. How many times per week do you consume vegetables. pasta. rice. or other dishes seasoned with sofrito (sauce made with tomato and onion. leek. or garlic and simmered with olive oil)?	≥2	83.30	86.50	0.738	81.80	92.30	0.226	88.20	88.30	0.558	100.00	82.60	88.90	0.470

Data are expressed as a mean (SD); ^a^ statistical significance *p* ≤ 0.05. Abbreviations: PAL, Physical Activity Level; tbsp, tablespoon.

## Data Availability

Data is unavailable due to privacy or ethical restrictions.
